# Engaging Adults Experiencing Homelessness in Recovery Education: A Qualitative Analysis of Individual and Program Level Enabling Factors 

**DOI:** 10.3389/fpsyt.2020.00779

**Published:** 2020-08-06

**Authors:** Bushra M. Khan, Nadine Reid, Rebecca Brown, Nicole Kozloff, Vicky Stergiopoulos

**Affiliations:** ^1^Centre for Addiction and Mental Health, Toronto, ON, Canada; ^2^Department of Psychiatry, University of Toronto, Toronto, ON, Canada; ^3^Centre for Urban Health Solutions, St. Michael’s Hospital, Unity Health Toronto, Toronto, ON, Canada; ^4^Institute of Health Policy, Management and Evaluation, University of Toronto, Toronto, ON, Canada

**Keywords:** recovery education, recovery college, homelessness, key ingredients, contextual features

## Abstract

**Purpose:**

Recovery Education Centres (REC) in mental health offer a new model of providing recovery supports through emancipatory adult education and recovery-oriented service principles. Despite the widespread adoption of RECs, there is limited evidence regarding factors enabling engagement and participation, particularly for unique subpopulations or service delivery contexts. The Supporting Transitions and Recovery Learning Centre (STAR) in Toronto, Ontario is the first REC in Canada and one of few worldwide supporting adults transitioning out of homelessness. This research aimed to investigate individual and program level enablers of engagement and participation in a REC for this population.

**Methods:**

Qualitative methods were used to explore the experiences of 20 service user participants through semi-structured interviews exploring their experiences of REC participation and perceived key program features. Interviews were conducted between July 2017 and June 2018, six to 14 months following REC enrollment, and analyzed using inductive thematic analysis.

**Results:**

In contrast to past experiences with health and social services, participants described a welcoming and respectful physical and interpersonal environment with low-barrier seamless access facilitating their engagement and participation. Although the realities of homelessness presented barriers for some, participants described that the involvement of peers, as role models, and the self-directed, strengths, and skills-based curriculum, co-produced and co-delivered by peers and professionals, were instrumental in activating the process of recovery through education.

**Conclusions/Implications:**

Findings are consistent with the growing evidence base of the defining features of RECs and suggest this model can be successfully extended to support recovery among adults transitioning out of homelessness. This unique examination of Canada’s first REC for adults exiting homelessness can help guide program and policy development to better support this disadvantaged population.

## Introduction

Homelessness and housing insecurity are increasing challenges in many jurisdictions (1). The city of Toronto, Canada’s largest urban center, is expanding at the fastest rate in North America (2) and has the largest homeless population in Canada (3). A 2018 point-in-time count identified 7,154 homeless people in the City of Toronto, nearly twice as many as the previous 2013 count (4). Homelessness has been associated with a higher burden of chronic health problems, mental health challenges, substance use, cognitive impairment, and consequently, premature mortality, increased acute care use as compared to the general population (5) higher health care costs (6, 7). Furthermore, despite experiencing a higher disease burden, homeless, and precariously housed populations experience stigma and discrimination in health settings and have poorer access to primary healthcare (8–10). The increasing numbers of homeless people, health inequities, and poor access to appropriate services have brought the issue of homelessness to the forefront in recent years (1).

Among health conditions, the prevalence of mental health and addictions problems and illnesses in homeless populations is alarmingly high (11). Several interventions have been evaluated in efforts to improve outcomes among people experiencing homelessness and serious mental health conditions, including assertive community treatment, intensive case management, critical time interventions, and supportive housing interventions such as Housing First (12–14). Although these interventions may impact housing stability and health service use, recent research has highlighted that the evidence on interventions that improve recovery outcomes for this population is scant.

In recent years, recovery has become a new paradigm and target for intervention within mental health services, and in the community. Mental health recovery is defined as a process of personal change leading to a satisfying, hopeful, contributing life, even within the limitations of mental illness (15, 16). Recovery-oriented service models complement traditional health services, emphasize the strengths and resources of people with mental illness, and focus on enhancing choice, control, and self-determination (17, 18). As with any new approaches, there have also been critical perspectives of recovery with major critiques arising from misunderstandings regarding the tenants of recovery. Recovery is not about independence and contribution to society when one has fully recovered, but rather about inclusion, and capacity to accommodate and accept the spectrum of individual abilities, regardless of where they are in the process of mental health recovery (18).

Among recovery-focused interventions, Recovery Education Centres (RECs) offer recovery supports through education rather than traditional health and social services (15). First established in the United States in the 1990s, RECs use an adult learning approach rooted in collaboration between service users and providers, working together in co-production, co-delivery, and co-learning (19–21). Recovery Education Centres now operate in over 20 countries, including the United States, Canada, Australia, New Zealand, and Europe (22), with the highest number in the United Kingdom, which currently has over 85 RECs (23, 24).

Despite the rising popularity of RECs, and progress in defining key ingredients and fidelity measures (25), there remains wide variation in implementation in practice and little research on REC adaptations for diverse subpopulations and service delivery contexts. Given the paucity of research on interventions that improve recovery among people experiencing homelessness and mental health challenges, this study of Canada’s first REC for individuals transitioning out of homelessness uses qualitative methods to examine factors supporting the engagement and participation of this population in recovery education. In particular, through participant narrative interviews, the study exposes individual and program level enablers of engagement and participation, and key REC features activating the process of recovery.

## Methods

The Supporting Transitions and Recovery Learning Centre (STAR) was the first REC in Canada and the only REC in North America with the mandate of supporting individuals transitioning out of homelessness (26, 27).

### Intervention

At STAR, members participated in classes of their choice led by peers with lived experience of homelessness and mental health challenges, as well as social and health service providers, within a curriculum co-developed with service users (26). Individuals were referred to STAR by other service providers or were self-referred.

STAR employees, overseen by a program manager, included a team leader and an administrative assistant, as well as five peer specialists, employed in a part-time capacity, from 0.2 to 0.6 of a 40-h full-time equivalent work week. STAR’s caseload was between 60 and 80 unique service users at any one time, with the majority of individuals taking one to two courses per semester.

The center operated as a “hub-and-spoke” model where the hub is a community center with a central classroom and staff offices (28) in the Regent Park neighborhood of Toronto and spokes included courses offered at partner organizations including public libraries, employment centers, and art studios. Course topics included health and wellness, vocational skills, leadership and community engagement, hobbies and interests, and life skills related to transitioning from homelessness to housing. Courses were held Monday to Friday from 10 am to 12 pm and 1 pm to 3 pm with a noon lunch program offered at the hub. The program was funded through a charitable donation from the Urban Angel Program for Homeless People through St. Michael’s Hospital.

### Study Design

This study is part of a rigorous mixed methods quasi-experimental evaluation of the STAR Learning Centre, located at St. Michael’s Hospital in Toronto, Canada. Qualitative methods were used to explore the experiences of participation of homeless and precariously housed participants (26). A realist-informed interview guide explored participants’ perspectives on factors allowing for engagement and participation and key REC ingredients supporting the process of recovery through education. Research Ethics Boards at St. Michael’s Hospital (Reference Number 16-179) and the Centre for Addiction and Mental Health approved this study (Reference Number 089/2016).

### Eligibility

STAR participants were 16 years of age or older, and had experienced challenges maintaining housing within the past 2 years. Furthermore, study eligibility criteria included: i) capacity to provide informed consent for research participation; ii) being a new member of STAR during the study recruitment period; iii) completion of at least 10 h of STAR classes (26).

### Data Collection

Of the 92 participants in the intervention arm of the larger study, 23 were recruited through purposive sampling to participate in an in-depth, in-person, semi-structured interview. Participants were selected based on willingness to participate, and ability reflect on and provide insights into their experiences with STAR as well as other services. Recruitment took place between July 2017 and June 2018, 6–14 months following program enrollment, and continued until data saturation was reached and no new themes emerged (29). Of the initial 23 participants recruited, 20 (or 87% of total recruited) consented to participate.

A semi-structured participant interview guide was created during the research protocol development phase (26). Key topics explored included motivation for enrolment, experiences with STAR and other services, key program components, as well as mechanisms of change and participant outcomes.

All participants provided written informed consent to participate. Participants received a $30 honorarium and two transit tokens for each interview. All interviews were recorded with participant permission and transcribed verbatim.

### Analysis

Qualitative interview and focus group data were analyzed through an inductive thematic analysis approach (30), using QSR International NVivo 9 software (NVivo, RRID : SCR_014802) (26). Three research staff coded seven transcripts independently to develop a set of key concepts or “codes” and compared findings. The codebook was developed incorporating feedback from all coders and the principal investigator. Once consensus was achieved, all transcripts were coded by the same three researchers. Nine additional transcripts were coded in the first two rounds of coding, with three transcripts double-coded, findings compared, differences resolved, and consensus achieved. In the final round of coding, four transcripts were coded, with three double-coded to establish inter-rater reliability (k=0.72) (26).

Some codes, related to potential key ingredients and other key program characteristics were identified based on literature reviews, using a deductive approach, while the majority of codes, themes, and subthemes emerged inductively during analysis of the transcripts. During analysis, similar codes were grouped into a set of high-level themes supported with direct examples and quotations from the sources. The research team discussed the categories, collectively reduced them to a smaller set of higher-level themes and refined them through an iterative process of review and feedback (30) to enhance analytical rigor. A checking process was completed with two staff members, including a peer specialist, to validate accuracy of the findings. At the time of writing this paper, STAR members could not be reached to participate in a member checking process.

## Results

Participant interviews lasted from 35 min to 100 min. The mean hours of STAR participation among study participants was approximately 80 h. At the time of their interview, the mean age of participants was 44.6, the majority identified as female (N=13 or 65%) and Caucasian (N=16, 80%). Further demographic characteristics of participants are shown in **Table 1** (31).

**Table 1:** Demographic characteristics of REC participants (N=20).

**Characteristic**			**Value**
Number of hours of program participation [mean (SD); median (IQR)]			80.4 (50.8); 66.8; (73.1)
Gender [n (%)]		Male	7 (35%)
		Female	13 (65%)
Age at time of interview in years [mean (SD); median (IQR)]			44.6 (12.5); 49.0 (19.0)
Education level (n (%))	High school or less		5 (25%)
	Some post-secondary, including university, business, trade or technical school		4 (20%)
	Completed post-secondary, including university, business, trade or technical school		11 (55%)
Ethnicity [n (%)]	Caucasian		16 (80%)
	Non-Caucasian		5 (20%)

Participants described largely negative past experiences with local health and social services, highlighting challenges in accessing needed services and revealing personal contextual factors that made engagement in recovery challenging for some. Participants’ perspectives and experiences with STAR were overwhelmingly positive, with no negative experiences emerging as a theme. Participants further highlighted factors that facilitated engagement and participation in STAR, such as the low barrier access and the welcoming and respectful physical and interpersonal environment. Finally, participants described defining program characteristics supporting their engagement in the process of recovery, including the involvement of peers, as role models, and the self-directed, strengths, and skills-based curriculum, co-produced and co-delivered by peers and professionals. These themes and subthemes are further detailed below.

### Contextualizing Experiences: Contrasting Recovery Education Experiences With Those at Other Services

Most participants had extensive prior service use, including emergency shelters, housing services, hospitals, mental health assistance and drop-in programs. This allowed for rich descriptions of experiences with both STAR and other services, as described below:

#### Recalling Past Encounters With Health and Social Services

The majority of participants (n=15) reported few past positive experiences with services, highlighting a lack of availability of needed services, lack of awareness of existing services, extended wait-times, time-limited availability, and services that were not geared to their health needs. As one participant described:

I went in there when I was homeless and when I got there they said, “We won’t be able to give you an appointment for another month.” So, like, so what do you do here? … Who knows if it was out in the winter you’d be out in the cold for a month, and they have no alternative. (P62)

Participants also highlighted the lack or responsiveness of services, as this participant described:

[I] put myself back into emergency, again was observed for seven hours and released again, with the promise that I would get a follow-up within 2 weeks for counseling. The call never came. I called them three weeks later, [they said] “Well we don’t do that anymore”. So, I was basically [abandoned]. (P78)

Other participants described that available services were not suited to their needs, or were experienced as stigmatizing:

[l]ook a certain way, or … look more challenged … if you don’t reek of piss, they kinda don’t talk to you. Some services I find are like that and how clean or dirty do I have to be to get my needs met. (P114)

#### Overcoming Barriers, Engaging With STAR

##### Individual Factors

Many participants described how their personal circumstances hindered their full engagement with STAR and other services. The majority of participants endorsed barriers such as active physical health, mental health, and/or addiction challenges (n=13), while several highlighted the lack of social capital such as family supports or education (n=8). Others described the impact of precarious housing on their ability to focus on participation and recovery (n=9). One participant discussed turning to alcohol to manage their stress: “two weeks into being homeless I started drinking, daily. I’m a daily drunk when I drink … life is just easier so to speak” (P1). Others described wishing they had the support of others: “I had some family members, but again, being judged from “It’s all your fault, you should’ve been more conscious about stuff” (P108) while another participant considered if their outcome would have been different had neighbors been supportive: “maybe had they been friends I would have gotten a weekend on a couch or something” (P1).

##### Program Factors

Notably, participants described overwhelmingly positive experiences with STAR, and described program characteristics facilitating engagement and participation. They highlighted the importance of the attractive and dignifying physical space, the low barrier access, the seamless experiences with registration and enrollment, and the welcoming interpersonal environment. As this participant commented: “I remember being like I don’t know what I’m doing, I’m nervous but this community center is gorgeous, and I’ve always wondered about it” (P109). Another participant shared:

[S]he gave me the right contact [information], there’s no wait list to get in, …. There wasn’t all this form you had to fill in, there’s no intimidation, it’s all straightforward. [I was] totally supported (P160).

Participants alluded to ownership being established in the enrollment process: **“**you have to actually physically enroll, and that’s what makes it different compared to other programs that I’ve been at” (P63).

The majority commented on feelings of support and belonging fostered by the welcoming and hospitable nature of staff and co-participants. One participant stated, “I belonged somewhere” (P109**)** and another described being listened to and treated with kindness, justifying their continued engagement: “I feel really listened to, really cared about” (P166).

#### Engaging in the Process of Recovery: Perceived Key Ingredients

Several valued program characteristics were highlighted by study participants as key ingredients facilitating engagement in the process of recovery, including involvement of people with lived experience in every aspect of the REC; participatory processes; and an individualized and skills-focused educational curriculum.

##### The Value of Lived Experience

Histories of mental health, addiction, and/or housing challenges were openly shared at STAR, and nearly all participants (n=18) strongly valued lived experience being part of every level of the program. Participants explained that “[b]ook smarts is great but book smarts plus an experience is a little bit stronger” (P133) because “I know that these people have the experience, so they know what they’re talking about” (P136). Specifically, participants believed the inclusion of staff and volunteers with lived experience made the program feel more relevant, practical, approachable, and effective. As one participant described,

[i]t makes the program a lot more honest, a lot more relevant, a lot more down to earth, when you’ve got someone like the program director willing to share his struggles when he was younger with addiction … and homelessness … just adds a huge amount of legitimacy to the whole program … Makes it, in my mind, ambitious, inclusive. (P115).

That is, the level of participant engagement, support and connection was bolstered by a culture at STAR where experience with mental health challenges was both valued and shared. For some participants, this program feature was instrumental to engaging them in the model:

[T]he teachers, the peers support workers also coming from lived experience … it is not a clinical approach or a therapeutic approach. It’s one of facilitation and support that I would find very hard to achieve in a therapeutic or clinical setting. The power and value of the lived experience … is invaluable … I believe it’s mandatory in how effective STAR is. (P78)

As another described: “[i]f you don’t abide by their curriculum then you’re perhaps not accepted to certain programs, whereas with STAR, they’re open to those things and help people with that in this very relatable way” **(**P88).

##### Participatory Processes

Nearly two thirds of participants (n=13) spoke about the participatory approaches to teaching and learning at the center. Participants valued that programming was developed and delivered in an inclusive way, such a way that “there’s no hierarchy” (P160) and “they [program staff] never feel like they are superior” (P1). Participants described a “very nimble, very open environment for making suggestions” (115) and valued opportunities to become involved in content design and delivery *via* facilitation.

Feeling their voices were heard, and opinions were respected within the program was important to participants as they explained how regular town hall meetings were held during which they were encouraged to contribute to conversations about proposed programming changes. As one of the participants further described,

[e]verybody had an opportunity to give feedback or input … they ask for feedback on how they can improve the class and what kinds of things you think you want to learn … they’re always concerned about your feedback too to make it better. (P63).

Others described overcoming a fear of failure by participating as a facilitator through STAR: “it’s [okay] to fail … I felt really welcomed … I’ve been given the chance to facilitate classes which gives me that, you know, my self-esteem is right back up there” (P108).

##### Individualized, Skills-Focused Curriculum

Over half of participants (n=11) described their ability to self-direct their program participation and overall recovery as a defining feature. Participants described Individual Learning Plans as a key aspect that fostered their “self-direct[ion]” (P62) and “self-leadership” (P66). These plans created recurring opportunities to “make the choices that I want to make and when I say, ‘Hey, I want to follow this path,’ or ‘I want to get to this point’…they’re able to show me which courses I can do and I can choose whether I want to do them or not” (P109); and because “you have a say in it … you feel in charge” (P88). Participants used their learning plans as tools to develop their self-determination, self-management and keep their goals on track; for example, “always start[ing classes] off with ‘Why do you think you’re here’ or who I want to be today, not tomorrow” (P50).

Similarly, participants spoke about the “well-rounded curriculum based on the variable needs of the members” (P78), and the “different types of [courses] … different things to do…” (P89). As another participant explained,

[I]n our goal plan … we say what we need and … they find courses that fit that for us. Say you want to learn more about feelings and emotions and triggers, they will add that in. They will add in an art group. They will try to bring someone in for writing. (P50)

Others described how the curriculum enabled specific career goals, for example: “I’ve chosen the peer support route and they have those courses to achieve the peer support that I want to achieve” (P114). This STAR feature underscored contextual fit: “[i]t’s just a better fit than a lot of other things that I’ve tried…… it’s about learning” (P113) and [the program] was “very appropriate and supportive in what my goals are” (P62).

## Discussion

Despite the widespread adoption of REC worldwide, there has been little research examining key features of RECs in diverse service delivery contexts, or diverse subpopulations of people experiencing mental health challenges. This is the first study to examine service user perspectives and experiences with a REC for people experiencing homelessness and mental health or addictions challenges.

Our findings expose past experiences with services for this population in the local context and highlight key enablers of engagement and participation in recovery education. Findings also highlight valued key features perceived as instrumental in facilitating the process of recovery.

With multiple past negative experiences with services, and in the context of challenging personal circumstances, engaging in recovery education was not smooth for some participants, who struggled with the realities of homelessness, few social supports, and other personal barriers. These findings are in line with previous literature with participants attesting to physical and mental health challenges, homelessness, and precarious housing in addition to poor connections to the healthcare system as being experiences in their journey to recovery (32, 33). The literature further suggests that additional barriers to engagement with services for homeless populations include stigma, limited opportunities for gainful employment that may result from participation, and fears of not being taken seriously or not feeling worthy enough to participate (10, 34–38).

Participant narratives also described valued program features facilitating engagement and participation, including the open and inviting programming space and the streamlined enrollment and registration process. These findings are consistent with previous literature, highlighting that participants were not engaging with recovery programming that occurred in an unwelcoming environment (39–41). Participant narratives further support that the opportunity to set goals and the positive interpersonal environment fostered belonging and participation. The specific need for a supportive interpersonal environment has been discussed, to a limited extent, in other studies, highlighting the value of tailored, individualized services that are physically and interpersonally accessible, timely and responsive (34, 35, 42).

Similar to previous work with RECs in the UK, participants highlighted several key ingredients facilitating the process of recovery, including the involvement of individuals with lived experience of homelessness in the design and delivery of services; a participatory approach to teaching and learning; and an individualized, skills-focused curriculum, which emphasized self-determination (25). The key ingredients elucidated by this population experiencing homelessness or precarious housing support are supported by previous research by Toney and colleagues (25). Other recent UK studies have indicated that the process of recovery occurs in the context of a level playing field (43), while more recent findings have emphasized that key ingredients of RECs include peer and professional involvement in the design and delivery of services through the process of co-production (44), a community focus, and inclusivity (16, 45).

The engagement of individuals experiencing homelessness and mental health and addiction challenges in the process of recovery could be mapped onto the Transtheoretical Model of Change (46) with individuals moving through precontemplation, contemplation, and preparation prior to engaging with recovery education. This model then depicts individuals working toward action and maintenance of growth in alignment with the CHIME recovery framework, a conceptual model for personal recovery in mental health consisting of 5 recovery processes: connectedness, hope and optimism about the future, identity, meaning in life, and empowerment (16). As outlined in the CHIME framework, participant interviews reflected increased connectedness, sources of support, and feelings of being part of a community (16).

Finally, this study exposed, similar to prior research with this population, critical areas where current programming in the local context is failing. Participants reflected on limited service availability and accessibility, long wait times, and time-limited service provision or inappropriate services. Given the complex health needs and poor health and social outcomes of this population, efforts to promote housing stability as well as recovery are urgently needed. Additional research is needed to guide policy and practices that consider and address the structural barriers and the broader social context in which Recovery Education interventions operate, particularly for socially disadvantaged populations.

### Strengths

The individual REC described in the current study has fidelity to the overarching REC model of care and consequently, findings of factors facilitating engagement and participation and key program features supporting the process of recovery may be generalizable to other recovery colleges and settings (26, 44). Furthermore, our study is unique in its focus on a population experiencing homelessness or precarious housing and highlights individual and program level factors facilitating engagement of a hard to engage population.

### Limitations

Study limitations include the point-in-time observations and as a result, do not provide a long-term perspective of how program features impact longitudinal experiences of recovery education. Furthermore, the key ingredients discussed were elucidated from a single study site; multiple settings would be ideal to deduce and concretize key program ingredients for this population. Other limiting factors include the characteristics of our sample, including participants who were predominantly women, Caucasian, and had some post-secondary education. These characteristics may not be representative of the larger population of individuals experiencing homelessness in different settings, or of STAR program participants. STAR service users were ethnically diverse with slightly more male (56%) than female service users; the STAR hub was also located in an ethnically diverse neighborhood. It is possible that participants from diverse backgrounds were reluctant to participate in this component of the research study or faced additional barriers to participation. Finally, although people with lived experience of homelessness contributed to the study protocol and interview guides, and gave early feedback on emerging themes, they did not participate in drafting or revising this manuscript.

## Conclusions

The study exposes key contextual factors affecting engagement with recovery education, and perceived key ingredients of a Recovery Education Centre for people experiencing homelessness and mental health challenges. Findings can inform efforts to enhance health and housing interventions and improve recovery outcomes for this disadvantaged population.

## Data Availability Statement

The raw data supporting the conclusions of this article will be made available by the authors, without undue reservation.

## Ethics Statement

The studies involving human participants were reviewed and approved by St. Michael’s Hospital and the Centre for Addiction and Mental Health, Toronto, Ontario, Canada. The patients/participants provided their written informed consent to participate in this study.

## Author Contributions

BK led the data analysis and manuscript preparation. NR participated in data analysis and drafting of the manuscript. RB participated in data collection, data analysis and drafting of the manuscript. NK participated in drafting of the manuscript. VS secured funding for the study, developed the research protocol, participated in data analysis, and co-led drafting of the manuscript. All authors contributed to the article and approved the submitted version.

## Funding

This study was funded by the Canadian Institutes of Health Research (grant number: 201409FDN). The funders had no role in study design, data collection and analysis, interpretation of data, or in writing of this manuscript.

## Conflict of Interest

The authors declare that the research was conducted in the absence of any commercial or financial relationships that could be construed as a potential conflict of interest.
