# Supplementary material for: Engaging Adults Experiencing Homelessness in Recovery Education: A Qualitative Analysis of Individual and Program Level Enabling Factors
Source: Front Psychiatry. 2020 Aug 6;11:779. doi: 10.3389/fpsyt.2020.00779 (PMC7424067; doi:10.3389/fpsyt.2020.00779)
Supplement: Supplementary file 1 [file DataSheet_1.docx]

**Supplementary Material**

**Appendix A. Qualitative Interview Guide (Intervention Group Participants)**

**Interview Guide: INTERVENTION PARTICIPANTS**

**[Review consent form with participant prior to commencing interview]**

*Do you have any questions before we get started? I’m going to start the recorder now – is that still okay with you?*

1. **Motivations for Enrolment at STAR Learning Centre**
2. I’d like you to tell me about the services, supports, or community organizations that you have used while living in a shelter or other unstable housing

- What types of services and programs have you used to support your recovery?

1. Could you describe your experience receiving support from other programs and services?

- What were the types of services that you found to be most helpful? Least helpful?

1. Why did you choose to enroll in STAR?

- Did you have a specific goal in mind? If so, what was it?

1. What were you hoping to get out of STAR that that you hadn’t gotten from other programs and services?
2. **Identifying Key Program Components of STAR**
3. Tell me about what it’s been like to be a member at STAR.

- What is the learning environment like?

1. How does the program support you and your recovery?

- How does it meet your needs?

1. *Choice***:** When it comes to your learning at STAR, do you feel like you’re in charge?

- How does the program help or hinder your ability to make choices about your own needs/priorities?

1. *Participation:* Do you feel included in decisions about STAR? Do you feel like your voice is heard when it comes to program changes?

- How does the program help or hinder your participation in these decisions?

1. *Coproduction:* What is it like to have peers, professionals, and members working and learning together in the program?

- How does it affect your experience at STAR?

1. *Peer involvement:* What is it like to have peer staff in the program?

- How does it affect your experience?
- Could you describe your relationship(s) with STAR staff?

1. *Links to services:* Do you feel like STAR has connected you to other services and resources?

- Which ones have been helpful? Which services or resources are missing?

1. *Adult ed. curriculum:* What are your overall impressions about the curriculum at STAR?

- Which classes have been helpful? Which classes are missing?

1. *Learning plans:* What are your thoughts on the Individual Learning Plan at STAR?

- How does it affect your experience at STAR?

1. What do you value most about STAR?
2. What would you most like to change about STAR?
3. How is your experience at STAR different than your experience with other programs? What makes it different?
4. **Program Outcomes and Mechanisms**

*In this section of the interview, I will ask you to reflect on the changes you may have observed in your own life after you started taking classes at STAR.*

1. *Stigma:* Has participating in STAR affected how you see yourself?

- Do you think it’s affected how others see you?

1. *Self-determination:* Has participating in STAR affected the level of control that you feel in your life?

- Do you notice a difference in your level of independence when it comes to making decisions about your life?

1. *Participation in desired activities:* Has participating in STAR affected your participation in other activities?

*[Probe:* e.g. education, employment or volunteering, or hobbies]

1. *Community integration:* Has participating in STAR affected your social connections with others?

- Has it affected your sense of community and belonging?

1. *Social functioning:* Has participating in STAR affected your ability to manage your day-to-day responsibilities

*[Probe:* *e.g. household, family/friends, work or volunteer tasks*]

1. *Self-management:* Has participating in STAR affected your ability to manage your mental health?

- Do you notice a difference in your ability to manage your symptoms, get information, and seek care when you need it?

1. *Wellbeing:* Has participating in STAR affected your sense of wellbeing (including mental, physical, social wellbeing)?
2. *Hope:* Has participating in STAR affected your outlook on your life?

- Do you notice a difference in how you feel about your recovery journey?

1. What are the biggest changes that you’ve experienced in your life since joining STAR?
2. **Transitions and Long-Term Recovery Outcomes**
3. What goals do you have in terms of your own recovery?

*[Prompt e.g. goals identified in your STAR Individualized Learning Plan]*

1. How has STAR helped you progress towards these goals? In what ways?
2. Have other programs/services helped you progress towards these goals? In what ways?
3. What other supports would you need, either during or after your time at STAR, to reach these goals?
4. We’ve now reached the end of the interview. Is there anything else you’d like to add?

Thank you very much for participating in this interview.
